# Towards better heartbeat segmentation with deep learning classification

**DOI:** 10.1038/s41598-020-77745-0

**Published:** 2020-11-26

**Authors:** Pedro Silva, Eduardo Luz, Guilherme Silva, Gladston Moreira, Elizabeth Wanner, Flavio Vidal, David Menotti

**Affiliations:** 1 Computing Department, Federal University of Ouro Preto, Ouro Prêto, MG 35400-000 Brazil; 2 Automation and Control Engineering Department, Federal University of Ouro Preto, Ouro Prêto, MG 35400-000 Brazil; 3 Engineering & Applied Science, Aston University, Birmingham, B4 7ET UK; 4 Department of Computer Science, University of Brasília, Brasília, 70910-900 Brazil; 5 Department of Informatics, Federal University of Paraná, Curitiba, PR 81531-990 Brazil

**Keywords:** Computational models, Data acquisition, Data processing, Machine learning, Network topology

## Abstract

The confidence of medical equipment is intimately related to false alarms. The higher the number of false events occurs, the less truthful is the equipment. In this sense, reducing (or suppressing) false positive alarms is hugely desirable. In this work, we propose a feasible and real-time approach that works as a validation method for a heartbeat segmentation third-party algorithm. The approach is based on convolutional neural networks (CNNs), which may be embedded in dedicated hardware. Our proposal aims to detect the pattern of a single heartbeat and classifies them into two classes: a heartbeat and not a heartbeat. For this, a seven-layer convolution network is employed for both data representation and classification. We evaluate our approach in two well-settled databases in the literature on the raw heartbeat signal. The first database is a conventional on-the-person database called MIT-BIH, and the second is one less uncontrolled off-the-person type database known as CYBHi. To evaluate the feasibility and the performance of the proposed approach, we use as a baseline the Pam-Tompkins algorithm, which is a well-known method in the literature and still used in the industry. We compare the baseline against the proposed approach: a CNN model validating the heartbeats detected by a third-party algorithm. In this work, the third-party algorithm is the same as the baseline for comparison purposes. The results support the feasibility of our approach showing that our method can enhance the positive prediction of the Pan-Tompkins algorithm from $97.84\%$/$90.28\%$ to $100.00\%$/$96.77\%$ by slightly decreasing the sensitivity from $95.79\%$/$96.95\%$ to $92.98\%/$ $95.71\%$ on the MIT-BIH/CYBHi databases.

## Introduction

False alarms may pose significant problems in intensive care units (ICU)^1^. Excessive false events lead hospital staff to lose confidence in such types of equipment, and thus, alarms from real problems may go undetected. The excessive false alarm problem has been extensively studied in the literature^1–7^.

**Figure 1:**
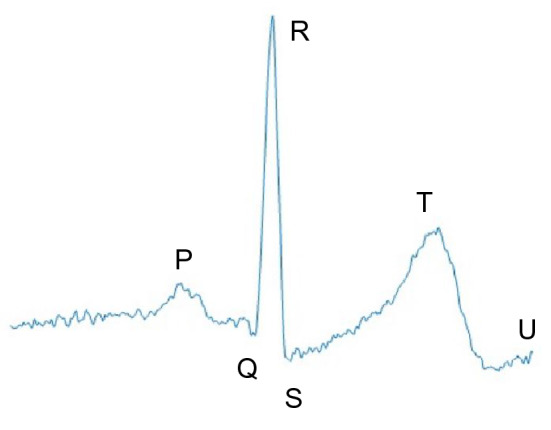
Fiducial points highlighted, plotted out over one ECG signal from CYBHi^11^ database.

Most of these approaches rely on the electrocardiogram (ECG) signal. The ECG is a vital signal, used to evaluate the electrical activity of the heart^8,9^. Its structure is composed of six fiducial points, presented in Fig. 1. Each fiducial point represents an event during the contraction/relaxation of the heart. Due to the information provided by this type of signal, ECG serves as the primary source for calculation of heartbeat rate and also for the detection and classification of cardiovascular diseases, such as arrhythmia^10^.

A competition considering the importance of the false alarm rate detection was promoted, the 2015 PhysioNet/CinC Challenge^12^. The 2015 PhysioNet/CinC Challenge was focused on five life-threatening arrhythmias. An arrhythmia can be described in two manners^13,14^: (i) a sequence of irregular beats, or (ii) as a unique irregular cardiac beat. Whether being harmful or not, the arrhythmia requests attention. Some cases need to be treated immediately, and in others, just a precautionary measure is required. For example, the tachycardia is one kind of life-threatening arrhythmia and needs to be treated immediately. From the works carried during the competition^12^, Plesinger et al.^3^ reported an outstanding score of 81.39% (the used competition score is a different weighted accuracy, i.e., $score = 100\cdot (TP + TN)/(TP + TN + FP + 5 \cdot FN)$). They proposed an approach based on the information of multiple channels, which were filtered by a cut-off frequency between 5 and 20 Hz, besides spectral features and descriptive residue statistics along with heuristic rules to achieve the reported metrics.

Arrhythmia detection is a straightforward application of the ECG signal. Therefore it relies heavily on the quality of the signal and also on the QRS detection algorithm (segmentation). Applications based on the ECG signal are commonly divided into four stages: pre-processing (filtering), ECG signal segmentation (QRS complex detection), signal representation using pattern recognition techniques, and classification algorithms. A failure in the segmentation stage propagates the error to the subsequent stages and directly affects the classification efficiency. Furthermore, the correct segmentation of the ECG signal and the identification of fiducial points are of paramount importance to reduce false alarms. However, many works in the literature^15,16^ focus on reducing false alarms in the classification stage, neglecting the error propagated by false alarms in the segmentation stage. Thus the motivation of this work arises: to reduce false alarms in the segmentation stage by using state-of-the-art pattern recognition techniques (a.k.a deep learning). It is important to note that convolutional neural networks (CNNs) have been applied to classify electrocardiogram (ECG) heartbeats in the diagnosis of arrhythmia^17–19^, which is a underlying subject to the scope of this work.

Several authors have worked on this problem (reducing false alarms during the segmentation stage), and one promising approach is the signal quality assessment, such as in Behar et al.^15^. The authors used machine learning to decide whether the signal is of good or bad quality. In according to Behar et al.^15^, the ECG signal is manually annotated in two classes (good quality signal and bad quality signal), down-sampled to 125 Hz, and seven quality indexes, which were used as a feature vector to train a support vector machine (SVM) classifier. The experiments were conducted in three databases, the Physionet, from the Computing in Cardiology (CinC) Challenge 2011^20^, the MIT-BIH arrhythmia database^21^, and the MIMIC II^22^ database. Behar et al.^15^ reported improvements in reducing false alarm for ectopic beats, tachycardia rhythms, atrial fibrillation, and on sinus rhythm.

Other authors employed a multi-modal approach, as in^16^, in which multiple ECG leads were used along with the invasive blood pressure wave. Quality indices, which resemble handcrafted feature extraction, were used in conjunction with a Kalman filtering algorithm. The method is evaluated with the MIMIC II^22^ database, and external noises were artificially added to the signals. Also, in^23^, a multi-modal approach is used to combine multiple ECG leads with pulse-oximeter (PPG) and arterial blood pressure (ABP) curves. A peak detection algorithm was proposed for each type of curve and improved by a quality assessment method. According to the authors, results showed a robust peak detection algorithm. The approach was evaluated on the Physionet Challenge 2015 database^12^.

In this work, a different approach is proposed based on deep learning techniques. The approach consists of a deep learning model validating the QRS complexes patterns detected by a third-party algorithm. Rather than relying on signal quality or the noise associated with it, we detect the ECG wave pattern, i.e., we detect (or validate) a heartbeat only by its shape. One advantage of this approach is to benefit from hardware accelerators for deep learning. Nowadays, there are many off-the-shelf deep learning accelerators, which means easy and effective integration with real equipment. Besides that, the proposed approach could be constantly improved by means of online learning. As the third-party algorithm, we select the well-known Pan-Tompkins algorithm^24^, since it is prevalent both in industry and academy. Moreover, it does not require significant computing resources. In summary, the main contributions of this work are:

- An efficient method for heartbeat pattern classification that operates in real-time to improve heartbeat segmentation.
- A CNN architecture for heartbeat classification.
- A proposal of a cyber-physical embedded system for heartbeat segmentation.

This work extends the one presented in the 23rd Iberoamerican Congress on Pattern Recognition (CIARP 2018)^25^ as follows:

- It presents an improved methodology, in particular, regarding the criterion for the selection of negative samples for training the deep learning model.
- It presents a more detailed evaluation and includes another challenging database (off-the-person category), i.e., the CYBHi database.
- It improved the experimental methodology by combining the CNN model with a popular QRS detection algorithm^24^.
- It adds a proposal to employ our approach in an embedded system context.

The obtained results show the effectiveness of the proposed approach to improve the QRS detection algorithms. Our approach enhances the Pan-Tompkins algorithm^24^ positive prediction from $97.84$ to $100.00\%$ in the MIT-BIH database and $91.81\%$ to $96.36\%$ in CYBHi. Though, there is a trade-off regarding sensitivity, and once there is a reduction from $95.79$ to $92.98\%$ in the MIT-BIH database and $95.86\%$ to $95.43\%$ in CYBHi.

In that sense, the proposed approach is feasible for real applications, since it allows the reduction of the false positive rate. The computational cost for the CNN inference has become increasingly attractive, since it is possible to embed the model in dedicated hardware, such as the Nvidia Jetson TX2 (available on https://developer.nvidia.com/embedded/jetson-tx2) and Field Programmable Gate Array (FPGA)^26^, for instance. This scenario facilitates the process of including this approach in Cyber-Physical/embedded systems, which is the case of medical equipment^27^.

## Methods

In this section, we present the methodology used to train CNN for ECG heartbeat recognition. Our method aims to validate the response of a well-known QRS complex detector from the literature. One may treat the QRS complex detector as an R-peak detection or heartbeat detection.

**Figure 2:**
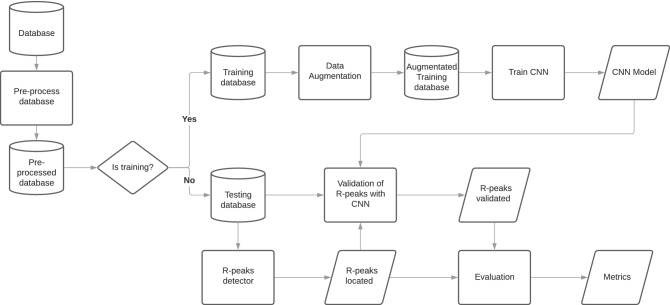
Proposed method flow.

The proposed approach is seen in Fig. 2 and can be divided into six main steps: (1) database split, (2) pre-processing, (3) train CNN, (4) R-peak detector, (5) validation of the R-peaks detected, and (6) evaluation. The database split is the process of separating it into train and test subsets. The pre-processing depends on the nature of the data and consists of dividing the original signal into several segments and apply data augmentation techniques. Step 3 is conducted using the training database to train a CNN. The R-peak detector consists of using some algorithm to detect the R-peak. The validation is given by authenticating whether the signal is a heartbeat (QRS complex) or not. In the last step (step 6), we report the metrics used to compare the algorithms.

### Database split and pre-processing

This step aims to divide the database into training and testing partitions. The first one is used exclusively to train a CNN and the latter for the testing phase. This process is necessary to avoid over-fitting and an overestimation of the proposed approach.

The pre-processing stage includes several steps and an adjustment of the input data size. Furthermore, since CNN requires a specific input size, all the segments must have a specific shape and a fixed sample window in time. Then, the input has been standardized to have 300 samples, in a 360 Hz sample frequency signal, resulting in 833 ms length. As a result, for a database sampled in 1MHz, the correspondent samples in 833 ms (833 samples) must be reshaped to 300 samples. Thus any 833 ms/300 samples-length segments are feed-forward into the network without any specific filter pre-processing.

### Data augmentation

This step also includes the application of data augmentation techniques for positive and negative samples. CNN benefits from this technique once it increases the amount of data and helps in convergence. Worth highlighting that, since CNN needs extra data only for training, we only apply the data augmentation techniques over the training sets.

According to Silva et al.^25^, to construct the positive samples, simple data augmentation is applied by considering the centralized R-peak and heartbeat signal shifted by exactly $\pm 5$ samples. For the negative samples, the heartbeat is shifted by exactly $\pm 30$, $\pm 50$, $\pm 80$, and $\pm 120$. A similar scenario presented in^25^ is considered: binary classification between segments with a heartbeat (positive samples) and without (negative samples). However, in this work, a different data augmentation approach is used to feed the deep learning model and this model applied with a different purpose. For the positive samples we use:

1. Centralized R-peak.
2. Shifted R-peak by ± 5 samples.
3. Shifted R-peak by ± 10 samples.
4. Shifted R-peak by ± 15 samples.
5. Centralized R-peak with P-wave (375 ms before the R-peak) attenuated by 30%.
6. Centralized R-peak with T-wave (375 ms after the R-peak) attenuated by 30%.
7. Centralized R-peak with a reduction of 20% over the entire segment.
8. Centralized R-peak with a reduction of 40% over the entire segment.

**Figure 3:**
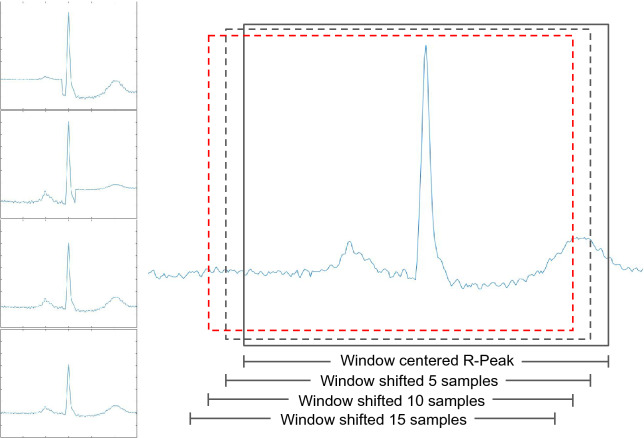
Example of the process applied to take the positive samples from a record. The waves plot in the left, represents, from top to bottom, the R-peak centralized with P-wave attenuated, T-wave attenuated, wave with attenuation about 20% and the last attenuation about 40%.

**Figure 4:**
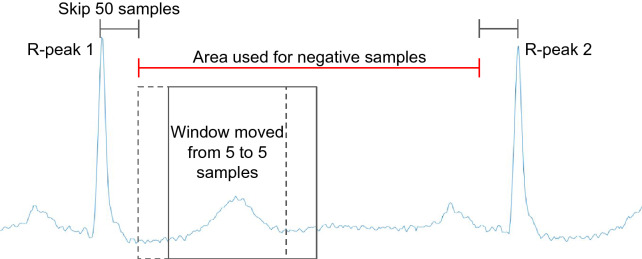
Example of the process applied to take the negative samples.

For the negative samples, all data between two R-peaks have used: 50 samples after the first R-peak and 50 samples before the second R-peak. This range marks the beginning and ending points of the sliding window, which is shifted by five-step stride (there is an overlapping among the samples within the two R-peaks). Figure 3 illustrates the data augmentation applied to the positive samples (sliding window, and wave manipulation) and Fig. 4 illustrates the construction of the negative ones. Since the QRS complex is the wave with the greatest amplitude within a heartbeat, it is less susceptible to noise. In contrast, the T and P waves have smaller amplitudes and usually a longer period of time and thus are more affected by all sources of noise. Thus, we propose a data augmentation attenuating the T and P waves, in order to force the model to be more immune to changes in the patterns of these waves.

### R-peak detector

In this step, a third-party algorithm is used to detect the R-peak along with the segment. Essentially, the QRS-detector method in this stage should be fast and have low computational power consumption. This stage is an essential step for our approach, once the amount of R-peak segments detected impacts on the time required by our approach to finish the process. For each R-peak detected, the CNN trained is used to infer if it is a real heartbeat or not.

The process starts with the ECG signal as the input for the R-peak detector. Then, this ECG signal is processed by the algorithm. At this point, each R-peak detector may apply a specific pre-processing that best fits its needs. The response of the method is the sample with an R-peak location. Some methods, such as the Pan-Tompkins^24^, may return a delay, which gives a range in where each R-peak may be.

In^24^, the authors designed the method using integer arithmetic, aiming a reduction in the computing consumption power to be as lowest as possible. A digital band-pass filter is applied by composing high and low-pass filters to reduce the impact of the noise over the signal, followed by one differentiation step and further a squaring step to intensify the slope and reduce the false-positives caused by the T waves. To detect the R-peak, Pan and Tompkins^24^ applied a sliding window along with an adaptive threshold, which results in an efficient and robust approach to discard noises. Therefore, it reduces the false-positive samples. To reduce the false-negative samples, those authors used a scheme with a dual-threshold, in which one is twice smaller than the other, and both have a continuous adaptation according to the current signal state.

Pan and Tompkins^24^ outlined a strategy based on the periodicity of the R-peaks on an ECG record. For the case in which an R-peak is not found within 166% of the current average interval, the maximal point in this interval, which lies between two thresholds, is considered as an R-peak, and as a consequence, a heartbeat or QRS complex. The authors highlight that this technique is only feasible for individuals with a regular heartbeat (without arrhythmia). For arrhythmic individuals, the authors proposed a reduction of both thresholds by half, to raise the sensitivity of R-Peak detection.

The authors also added two essential constraints regarding R-peak detection: (1) the next R-peak must occur at least 200 ms at a physiological point of view, and (2) an R-peak detection approach needs to adapt parameters to each patient continuously.

### CNN training

**Figure 5:**
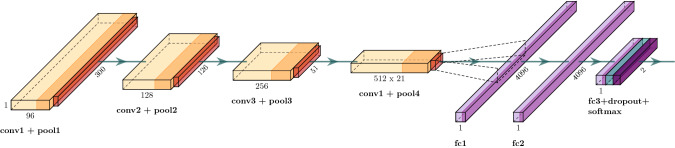
CNN used to validate the R-peaks^25^ in which the convolution layers *conv1*, *conv2*, *conv3* and *conv4* use filters size equal to 1*x*49, 1*x*25, 1*x*9 and 1*x*9, respectively, and stride equal to one. All pooling layers (*pool1*, *pool2*, *pool3* and *pool4*) uses max operation with filter size and stride equal to two. The padding is equal to zero for all convolutional and pooling layers.

The CNN model/architecture used here is the same used in our preliminary work^25^. It is composed of four convolutional layers, two fully-connected layers, a dropout layer to reduce over-fitting, and a final fully-connected layer with two neurons for binary classification: (1) this segment has an R-peak centered in the segment, and (2) segment without R-peak centered. Figure 5 shows such CNN architecture.

Different from our previous work^25^, in this work, the deep learning model is used as a second judge for a well-known R-peak detector algorithm. The present approach aims to enhance the result from the R-peak detection algorithm, aligned with state-of-the-art trends^28^.

Beforehand, to train CNN, a set of data is separated and labeled, usually by a human expert. This data is then used to generate positive and negative samples. Those samples are used to train the CNN as a simple binary classification problem: the output is a heartbeat or no heartbeat.

### Validation of R-peaks detected

In this step, any algorithm presented in the literature which aims an R-peak detection can be used. However, this step needs three inputs: an ECG signal, the R-peaks location detected by an R-peak detector algorithm, and a machine learning model. The output of this step is a set of all R-peaks locations in which the machine learning model agrees with the R-peak detector.

In this step, an 833-ms window centered in each R-peak detected is feed-forwarded through the CNN. The CNN confirms whether it is an R-peak in the center of the segment or not.

### Evaluation

To evaluate one database, a set of data is reserved as a testing partition. With the R-peaks validated by the machine learning model, the metrics used to compare the approaches are calculated. We compare the right and wrong detections of both approaches: the third-party algorithm by itself and the proposed approach, with the CNN as a validator. A correct heartbeat detection is considered when an R-peak is within the center of a segment with a tolerance of the shifts used in data augmentation described. A wrong detection occurs when an R-peak is not in this range.

## Results

In this section, the experiments are described in detail. Also, the results reached with the proposed approach are presented as well as the discussion.

### Experiment details

#### Database

To report the results presented in this section, we used two databases to train a CNN model: CYBHi^11^ (off-the-person) and MIT-BIH^29^ (on-the-person). To conduct fair experiments, we split both databases into two sets without patient intersection.

As the MIT-BIH database has a group of signals with healthy individuals and another group with individuals who have cardiac problems (arrhythmia), we decided to use only the first group to avoid impacts on the R-peak detectors algorithms and, therefore, in the final metrics reported. The healthy group has a total of 23 records, and each record received a numerical identification on the dataset. The records are: 100, 102, 104, 106, 108, 112, 114, 116, 118, 122, 124, 101, 103, 105, 107, 109, 111, 113, 115, 117, 119, 121, 123. All heartbeats (approximately 110,000) available with the MIT-BIH database have its R-peak location annotated by two cardiologists in a separate manner and disagreements were resolved by a third person. The annotations are available on the Physionet website. As CNN model benefits of more data, we decided to use the odd records to train and even for testing. We stress that each record belongs to a single subject and that there is no overlap of subjects on both training and test sets.

**Figure 6:**
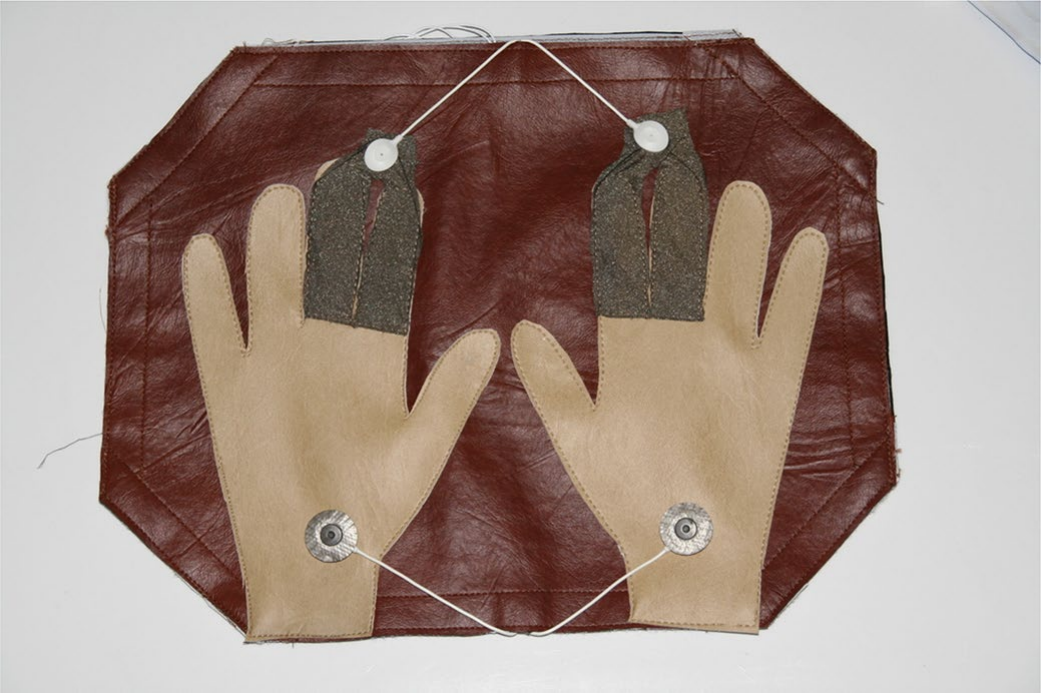
Equipment used to acquire the ECG signal employing the fingers. Source:^11^.

The CYBHi has more registers when compared to the MIT-BIH database with 126 records. As the database is captured with an off-the-person device, it suffers more with noise. The data acquisition is made using two differential lead electrodes at hand palms and fingers, as shown in Fig. 6.

**Figure 7:**
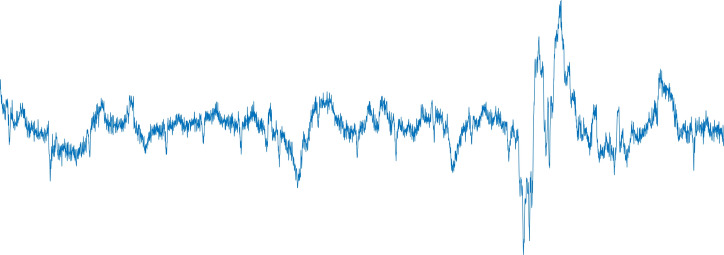
CYBHi disregarded signal/records: 10-s-sample.

We have discarded 12 records from the CYBHi because our specialists weren’t able to detect the heartbeats due to excess of noise. Therefore, we have no ground truth to train the model. Figure 7 presents a 10-s-segment which should have approximately 10 R-peaks, however, it is hard to detect them and, subsequently, label them. The remaining 114 records are used for training and testing. For the construction of the CYBHi database, the data acquisition happens in two distinct 2-min sessions with 63 subjects, into the range of 3 months.

For each one of 63 subjects, two sessions were acquired in two different setups: Short-term signals and Long-term signals. Only the latest one is used in this work since it is a more challenging scenario^30^. The CYBHi database’s authors did not provide the R-peak location annotation. Thus, this annotation was made by the researchers of this work and will be provided along with the source code. According to Luz and Menotti^31^, the data from a patient must be on the training set or in testing, not both. Upon this fact, we ignored the natural division of CYBHi database and used both sessions of an individual only to train the CNN or to test. We selected half of the subjects to training and half for the test set, randomly, and for reproducibility, the records are made available at https://github.com/ufopcsilab/qrs-better-heartbeat-segmentation.

#### Resulting data augmentation

Table 1 presents the total of training samples with and without Data Augmentation (see detail on how data augmentation is performed in “Methods” section). As one may see, the number of negative samples (No R-peak) is the same independently whether the Data Augmentation is used or not. The main difference is the number of positive samples (R-peak), which turns possible to train a CNN model.

**Table 1:** Total training samples with the presence or absence of the Data Augmentation used.

Data Augmentation	MIT-BIH		CYBHi	
	R-peaks	No R-peak	R-peaks	No R-peak
No	16,647	98,586	9414	47,110
Yes	183,117	98,586	103,554	47,110

To train the models, we allocate 70% of a register’s data (data of one individual) for the training partition and the remaining 30% to the validation partition which is used only for network optimization. Thus, from the total number of images presented in Table 1, 70% is used for training and 30% for validation. The list of records selected for both training (train/validation) and test partitions, for both databases, are available at https://github.com/ufopcsilab/qrs-better-heartbeat-segmentation.

#### R-peak detector

For this work, we evaluate our method with an implementation of the Pan-Tompkins^24^ algorithm as the third-party one for the R-peak detector. We used the *MATLAB* implementation available in^32^ to run our experiments.

#### CNN training

As CNN input size, we use 833 ms, which means a 300-sample-size for the MIT-BIH database and 833-sample-size for CYBHi. Both represent 833 ms of the record. Since the CNN input size is fixed, it is necessary to conduct a down-sampling of the CYBHi signal in order to keep the same network architecture. We use a polynomial interpolation to perform the down-sampling. The same offsets used for data augmentation described in “Methods” section are used for both databases (MIT-BIH and CYBHi).

**Figure 8:**
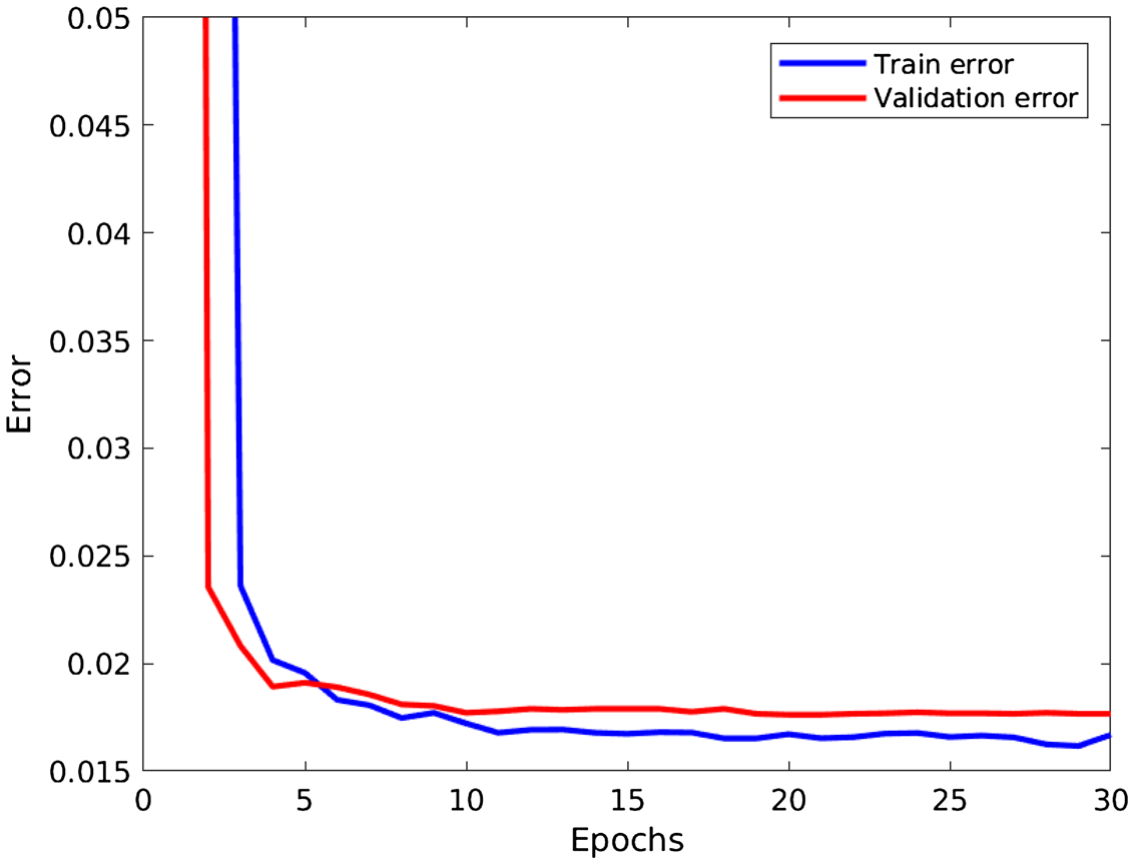
Train and validation error over the 30 training epochs on CYBHi database.

The CNN is trained for 30 epochs with learning rate equals to 0.01 for the first three epochs, followed by 0.005 for seven more epochs, 0.001 for another 10 epochs and, finally, 0.0001 for the remaining 10 epochs. Also, stochastic gradient descent with momentum (0.9) is used for network weights optimization, *softmax*-loss as the activation function and the binary-cross-entropy as the cost function. Figure 8 shows the train and validation over the 30 training epochs on the CYBHi database. As one can see, the train and validation error drops fast in the early epochs and stabilizes after five/ten epochs. Since in this training phase, we have data from the same patient (individual) both in the 70% data reserved for training and the 30% data reserved for validation.

#### Validation of R-peaks detected

The third-party algorithm Pan-Tomp-kins^24^ used as an R-peak detector returns two responses: (1) R-peaks detected, and (2) a delay. The R-peaks are the center of the R-peaks, while the delay defines a window in which the R-peaks may be located. Those detections may have some missing R-peaks, or even wrong R-peaks detected. Those wrong R-peaks could be harmful to real applications and should be discarded. The validation, with a pattern recognition model, can be a workaround for this issue. The validation occurs once the Pan-Tompkins algorithm finds an R-peak, and the output of the CNN model feed-forwarded agrees that it is a heartbeat.

#### Evaluation

The most common metrics for heartbeat segmentation methods are: sensitivity (Se) and Positive Predictive (+P)^33^. We also report F-Score as a harmonic average of *Se* and $+P$. The measures described are defined as:

$$+P_{Seg}= \frac{TP}{TP + FP} \quad Se_{Seg}= \frac{TP}{TP + FN} \quad F\text {-}Score_{Seg}= 2 \times \frac{+P_{Seg} * Se_{Seg}}{+P_{Seg} + Se_{Seg}}$$

We treat this problem as a binary classification, in which an R-peak detected is a positive class, while segments without R-peak information is a negative class. Based on this, True-Positive (TP) is a segment well detected, False-Positive (FP) is an erroneous segment detected as R-peak. The False-Negative (FN) is a right R-peak segment that is falsely discarded. It is worthwhile to note that our proposal can only improve the $+P$ along with a small degradation on *Se*.

### Analysis

In Table 2, the results are presented for both databases with the metrics already described. We compare the standard R-peak detector algorithm against our proposed methodology.

**Table 2:** Results from the proposed approach against Pan-Tompkins algorithm.

Database	Pan-Tompkins			Proposed approach		
	Se (%)	+P (%)	F-Score	Se (%)	+P (%)	F-Score
MIT-BIH	**95.79**	97.84	**0.97**	92.98	**100.00**	0.96
CYBHi	**96.95**	90.28	0.93	95.71	**96.77**	**0.96**

Results presented in Table 2 show the gain in positive predictive from the CNN as a validator. Nevertheless, a reduction in the sensitivity metric is perceived. However, the F-Score is maintained equivalent over the two databases. The figures representing our analysis are highlighted in bold in Table 2.

**Figure 9:**
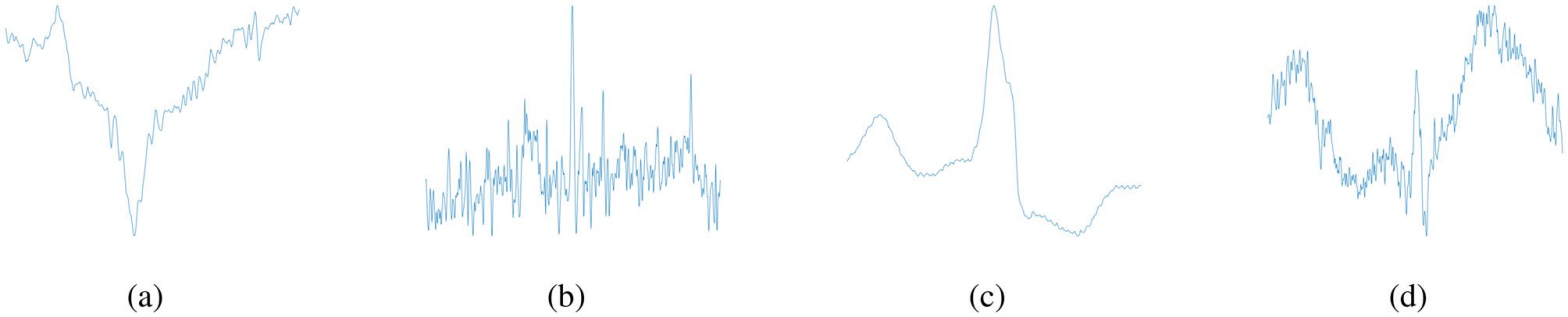
Examples to illustrate the effects of our proposal.

Our approach enhances the Pan-Tompkins algorithm positive prediction from $97.84$ to $100.00\%$ in the MIT-BIH database and $90.28\%$ to $96.77\%$ in the CYBHi. Although a reduction in Sensitive is observed in both databases, in which the Pan-Tompkins approach reaches $95.79\%$ and $96.95\%$ and our approach $92.98\%$ and $95.71\%$ for MIT-BIH and CYBHi, respectively. A reduction in the F-Score metric occurs in MIT-BIH, from 0.97 (Pan-Tompkins) to 0.96. While, in the CYBHi, the opposite occurs, once the F-Score enhanced from 0.93 to 0.96. In Fig. 9, we show examples to illustrate the effects of our proposal. The heartbeats in Fig. 9a,b are samples from MIT-BIH and CYBHi databases, respectively that were wrongly classified as FPs by the baseline approach and now are correctly classified (rejected) as TNs. Conversely, in Fig. 9c,d, we show samples from MIT-BIH and CYBHi databases that were classified as one true heartbeat (TPs) by the baseline method and corrected to Non-heartbeat (FN) by our approach. By increasing the positive prediction (diminishing the FP rate), the beneficial effects that our proposal promotes is to provide reliable samples to further analysis, such as arrhythmia classification. Contrasting to that, by diminishing the sensitivity of the heartbeat segmentation (increasing the FN rate), our approach may exclude true samples, which can be prohibited in some applications. Such trade-off should be adjusted according to the application.

**Figure 10:**
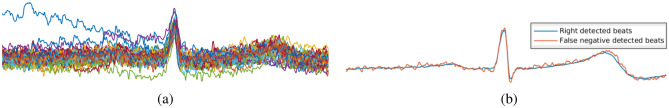
Analyses of CNN results in CYBHi database. In (**a**), all false-negative heartbeats of a specific subject are presented, and in (**b**) the means of the true-positive and false-negative missed beats of a register are presented. A true positive heartbeat detection means a segment with the R-peak centralized with a window size of 15 samples in both sides due the train protocol implemented. A false positive heartbeat detection means a segment in which the R-peak is not centralized.

Our hypothesis for the sensitivity reduction is the high-frequency noise altering the morphology of the signal. Our model relies on the morphology of the signal to determine whether the segment is a heartbeat or not. Thus, high-frequency noises alter the shape of the curve, especially the P and T waves, which are temporarily wide (see Fig. 10).

The Pam-Tompkins is a peak detection algorithm and does not rely on the signal morphology, different from our approach. If the morphology changes due to the high frequency, it has a negative impact on our approach, which is not seen in the Pam-Tompkins approach. The CYBHi database signal morphology is changed due to the noise, as seen in Fig. 10b, making heartbeat segmentation difficult. As the MIT-BIH database acquisition happened in a more controlled scenario, this problem is reduced, and the *+P* metric is greater than the CYBHi database.

As seen in Fig. 10a, it is possible to verify how abrupt the changes are in the signal of the same subject in the same record. A different perspective is presented in Fig. 10b, which shows the average variance of false-negative samples against the average true-positive (right detected) samples of a specific subject. This small average variance change impacts the final result, mainly in the sensitivity metric.

Based on the results in Table 2, one also may infer that the CNN architecture used is capable of generalizing and learning for both databases. The outstanding results confirm this hypothesis.

The popularization of deep learning, especially CNNs, has led to a fast increase in the development of specific hardware for inference acceleration. Thus, deep learning methods are an attractive option to be embedded in real products.

Once the deep learning model has passed the training stage, it can be used in inference mode (for production), which in our case means classifying a sequence of one dimension input sample as a heartbeat or not. The trained model can be embedded in hardware, and the inference accelerated with the aid of special circuits based on FPGA or GPU^26^. Today, GPUs still are state-of-the-art in inference throughput^26^.

**Figure 11:**
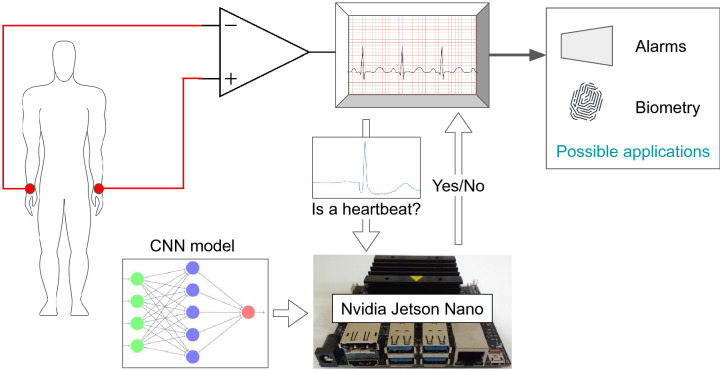
Example of the proposed embedded system in a representation of a real scenario with FV*NVIDIA Jetson TX2* Module. (Source: the authors).

In this work, we export our model to *TensorFlow* in order to allow compatibility with the *NVIDIA Jetson TX, TX2*, and *Nano* (see Fig. 11). The *NVIDIA Jetson Nano* board uses a 128-core Maxwell GPU and 4GB of RAM and can make inferences more than 20 times the speed of the most common CPU^34^. It facilitates the point in which the medical equipment^35^ can communicate with the board via USB bus, WiFi (TCP/IP), or even RS-232 standard, which favor the integration with real products.

In order to evaluate the computational cost (time consumption) of the proposed CNN, we repeat the inference process 100 times to evaluate the average time consumed by the network running in a CPU (*Intel* i7 8th generation), a GPU and an *NVIDIA Jetson Nano*. The total time consumed by the CPU is 3.291 s, with an average of 0.033 s, while in GPU, the total time is 1.001 s with an average equal to 0.01 s. In the *NVIDIA Jetson Nano*, one can observe a total of 3.339 s with an average of 0.033 s.

The *NVIDIA Jetson Nano* has equivalent performance to an Intel i7 with higher power efficiency, approximately 10 times less energy is required^36^. Furthermore, the worst-case scenario between two R-peaks is at least an interval of 200 ms^24^, which is greater than the proposed CNN model inference time required (33 ms average). Upon those facts, the proposed approach is a feasible scenario for the real world.

**Figure 12:**
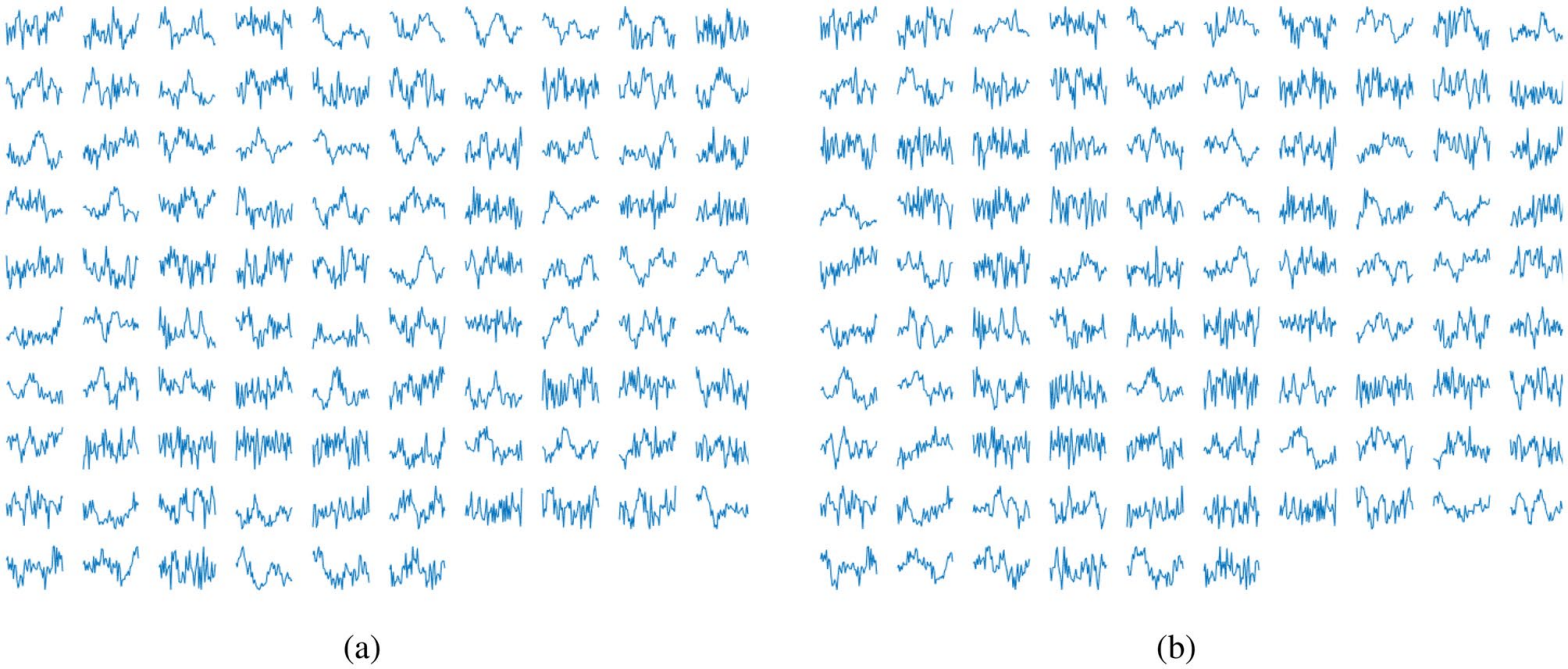
Outputs for the proposed CNN architecture for both databases. (**a**) First layer filters for a CNN trained in the MIT database. (**b**) First layer filters for a CNN trained in the CYBHi database.

Figure 12 presents the filters from the first layer of the proposed architecture for both databases, MIT and CYBHi. Both filters are initialized with the same seed. The filters are similar, but the filters from the CYBHi database (Fig. 12b) have a more extensive range when compared to the MIT ones (Fig. 12a). One of the possible reasons is due to the noisy nature of CYBHi signals.

**Figure 13:**
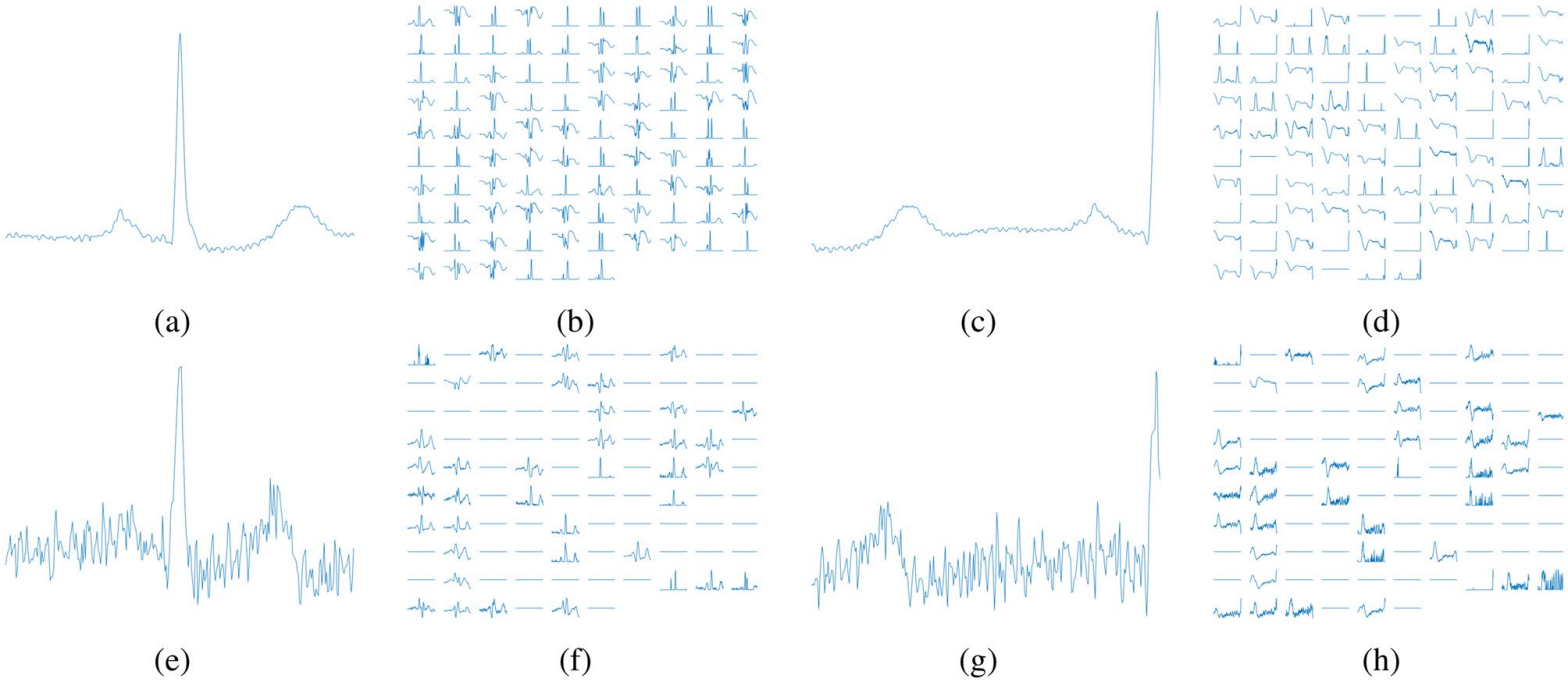
Filter outputs from the proposed CNN architecture. (**a**,**e**) are the inputs with a valid QRS complex from MIT and CYBHi databases, respectively. (**b**,**f**) are the outputs of the fourth layer from the signals presented in (**a**,**e**) and the proposed CNN architecture trained in MIT and CYBHi databases, respectively. (**c**,**g**) are the inputs with a not valid QRS complex from MIT and CYBHi databases, respectively. (**d**,**h**) are the outputs of the fourth layer from the signals presented in (**c**,**g**) and the proposed CNN architecture trained in MIT and CYBHi databases, respectively.

One can see how noisily are the signals from the CYBHi database in Fig. 13e,g when compared to signals of Fig. 13a,c from a controlled database, such as the MIT database. It is notable that several filters are sensible to a noisy ECG, as shown in Fig. 13f,h. Besides, the same behavior is observed in the output of the filters from the positive samples (Fig. 13b,f) and negative samples (Fig. 13d,h). In the first scenario, a peak is observed around the center of the activation map of the filters. For the latter scenario, the peak is located on the edges of the signal. The MIT-BIH DB has almost twice as many positive samples (QRSs) than the CYBHi database (see Table 1). With more data, the model learn better filters. Since the architecture is the same for both databases, the model trained with the CYBHi base suffers twice.

## Discussion

In this work, we proposed the use of a CNN aiming R-peak detection from a different perspective. Instead of using techniques based on a signal quality index, filters, or using other signals to validate the occurrence of a heartbeat (multimodal approach), we applied machine learning techniques, more specifically CNNs, to recognize the pattern of a heartbeat. Our proposal aimed to improve the detection of a traditional algorithm for R-peak detection and act as a validator method for R-peaks (or heartbeats). In that manner, we avoided a sliding window over the entire signal and, as a consequence, a reduction of the computation cost involving the entire machine learning inference process.

Since correct segmentation is critical for medical equipment, the positive prediction should be considered over the sensitivity. The reported results supported this scenario, in which our approach enhanced the Pan-Tompkins R-peak detector positive prediction on two distinct databases. However, it is worth highlighting that there is a trade-off between positive prediction and sensitivity. The low positive prediction could compromise the application by emitting wrong alarms, for instance. On the other hand, low sensitivity may result in a scenario where necessary alarms are not emitted.

One path for future work is the design and application of filters to avoid the high frequencies noises in the ECG signal, especially for off-the-person databases. Since the filter design needs an in-depth knowledge of the signal, a different approach is to apply a machine learning technique to learn which filter best fits for each signal. Another research path is the fine-tuning of a pre-trained deep learning model to enhance the generalization of the proposed approach without losing positive prediction.

The proposed method is trained to detect a pattern of a normal heartbeat. However, in a real environment, irregular or arrhythmic beats may appear and they may have a morphology completely different from the morphology of a standard QRS complex. Thus, another future investigation path would be to explore models capable of classifying other classes (types of heartbeat).

## Data Availability

*Source codes* https://github.com/ufopcsilab/qrs-better-heartbeat-segmentation.
